# Beyond Taste: The Impact of Chocolate on Cardiovascular and Steatotic Liver Disease Risk Factors

**DOI:** 10.3390/nu18040636

**Published:** 2026-02-14

**Authors:** Júlia Mayumi Tomaru, Iara Ribeiro Nunes, Caroline Fernandes de Souza Santiago, Alda Maria Machado Bueno Otoboni, Claudemir Gregorio Mendes, Adriana Maria Ragassi Fiorini, Elen Landgraf Guiguer, Claudia Cristina Teixeira Nicolau, Antonelly Cassio Alves Carvalho, Caio Sérgio Galina Spilla, José Luiz Yanaguizawa Junior, Vitor Engrácia Valenti, Ricardo de Alvares Goulart, Luiz Carlos de Abreu, Lucas Fornari Laurindo, Sandra Maria Barbalho

**Affiliations:** 1 Department of Biochemistry and Pharmacology, School of Medicine, Universidade de Marília (UNIMAR), Marília 17525-902, SP, Brazil; 2 Department of Biochemistry and Nutrition, School of Food and Technology of Marília (FATEC), Marília 17500-000, SP, Brazil; 3 Graduate Program in Structural and Functional Interactions in Rehabilitation, School of Medicine, Universidade de Marília (UNIMAR), Marília 17525-902, SP, Brazil; 4 Faculty of Philosophy and Sciences, Universidade Estadual Paulista (UNESP), Marília Campus, Marília 17525-900, SP, Brazil; vitor.valenti@unesp.br; 5 Faculty of Education & Health Services, School of Medicine, University of Limerick, V94 T9PX Limerick, Ireland; luiz.abreu@ufes.br; 6 Centro de Ciências da Saúde, Universidade Federal do Espírito Santo (UFES), Vitória 29075-910, ES, Brazil; 7 Division of Cellular Growth, Hemodynamics, and Homeostasis Disorders, Graduate Program in Medical Sciences, Faculdade de Medicina, Universidade de São Paulo (USP), São Paulo 01246-903, SP, Brazil; 8 Department of Research, Research Coordination Center, UNIMAR Charitable Hospital, Universidade de Marília (UNIMAR), Marília 17525-902, SP, Brazil

**Keywords:** chocolate, cocoa, antioxidant, anti-inflammatory, cardiovascular, liver diseases

## Abstract

Cardiovascular diseases and metabolic dysfunction-associated steatotic liver disease (MASLD) are increasing sharply worldwide and share overlapping pathophysiological pathways, including oxidative stress, inflammation, hyperglycemia, obesity, dyslipidemia, and hypertension. Dark chocolate, rich in cocoa flavanols such as epicatechin and catechin, exhibits antioxidant and anti-inflammatory effects. Based on these properties, this narrative review uniquely integrates evidence on chocolate’s effects on both cardiovascular and hepatic health, exploring shared mechanisms and clinical implications. Evidence from clinical studies suggests that chocolate modulates nitric oxide bioavailability and NADPH oxidase activity. Clinical findings demonstrate improvements in flow-mediated dilation, decreased NT-proBNP, reduced intestinal permeability and endotoxemia, improved lipid profile (increased HDL-c and reduced total cholesterol, LDL-c, and triglycerides), increased plasma polyphenols, improved platelet function, and attenuated hepatocyte apoptosis. These findings suggest a potential role for cocoa flavanol-rich dark chocolate in cardiometabolic health; however, the evidence remains preliminary and is limited by heterogeneous study designs, small sample sizes, and short intervention durations. Despite these limitations, current evidence supports the inclusion of moderate dark chocolate consumption as a possible adjunct strategy to mitigate cardiometabolic and hepatic metabolic risks. Further large-scale, long-term trials are needed to confirm these beneficial effects and to standardize the dosage and formulation of cocoa flavanols.

## 1. Introduction

Metabolic disorders are increasing sharply due to severe modifications in lifestyle [1,2,3,4]. These disorders may include metabolic syndrome (MetS), cardiovascular diseases (CVD), and liver diseases that are related to premature deaths. MetS is a multifactorial condition, with its primary indicators including obesity, hypertension, dyslipidemia, insulin resistance, and hyperglycemia; i.e., a patient who possesses at least 3 of the following parameters is designated with MetS: glucose intolerance, increased levels of triglycerides, augmented waist circumference, low levels of high-density lipoprotein cholesterol (HDL-c), and hypertension [5,6,7]. It is possible to develop a close relationship with MetS, metabolic dysfunction-associated steatotic liver disease (MASLD), and CVD [8,9]. MASLD disrupts the vascular endothelium, serving as an initiator of the atherosclerosis process. Furthermore, the oxidative stress and inflammatory process related to MASLD can contribute to the development of diabetes, lipid disorders, obesity, and hypertension [10,11,12,13,14].

CVD encompasses ischemic heart disease, stroke, heart failure, peripheral artery disease, and various other cardiac and vascular conditions [2,15,16,17]. CVD have collectively remained the leading causes of death worldwide and substantially contribute to loss of health and excess health system costs [18]. According to data from the World Health Organization (WHO), the leading cause of death worldwide is ischemic heart disease, accounting for 13% of all deaths around the world. Since 2000, the most significant increase in deaths has been from this disease, increasing by 2.7 million to 9.1 million deaths in 2021 [19,20,21,22,23].

MASLD is a growing global health concern [21,24]. MASLD is strongly associated with various conditions, including MetS, and is characterized by the persistence of a chronic inflammatory state detrimental to the organism [25,26]. Moreover, it is worth noting that recent global estimates indicate a prevalence of MASLD exceeding 30% among adults [27,28,29], underscoring the need for studies on this topic and its implications.

Diet and healthy lifestyle habits remain the most effective strategies for the general management of metabolic disorders, aiming to mitigate the effects of premature aging and mortality from causes associated with organ dysfunction [30,31]. According to the World Gastroenterology Organization (WGO), in MASLD and non-alcoholic hepatic steatosis, management focuses on addressing insulin resistance and oxidative stress. There is currently no approved pharmacotherapy for the entire spectrum of MASLD, and disease management remains largely focused on lifestyle modifications and the treatment of associated metabolic comorbidities. However, it is important to note that resmetirom has recently been approved by the U.S. Food and Drug Administration for the treatment of patients with noncirrhotic metabolic dysfunction-associated steatohepatitis (MASH) and moderate to advanced fibrosis, representing a significant advance for selected stages of the disease [6,32,33,34]. Therefore, disease management currently relies primarily on lifestyle interventions and on pharmacological treatment of associated metabolic comorbidities, such as hyperglycemia, dyslipidemia, and cardiovascular risk factors, rather than on drugs directly targeting the full MASLD spectrum [35].

Throughout human history, plants have been used as dietary components and sources of bioactive compounds with potential health-promoting properties. Traditional medicine has benefited from plant-derived products, and many modern pharmacological agents originate from natural sources. However, it is important to emphasize that, in the context of complex chronic diseases such as CVD and MASLD, evidence-based pharmacological therapies remain the cornerstone and standard of care, particularly in patients with established disease. Plant-derived compounds and functional foods should therefore be considered as complementary or adjunctive strategies that may support metabolic and cardiovascular health, rather than as alternatives to guideline-recommended medical treatments [36,37,38,39,40,41].

Cacao (*Theobroma cacao*) and chocolate have demonstrated several health benefits, such as reducing blood lipids, blood pressure, and endothelial dysfunction, as cacao contains compounds, including flavanols (epicatechin, catechin, and procyanidins), that exhibit antioxidant and anti-inflammatory actions (Table 1) [42,43,44,45].

**Figure 1:**
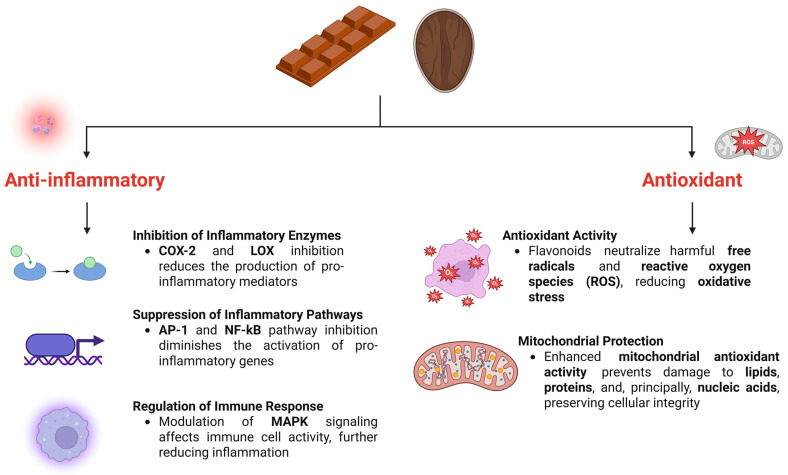
Effects of Cocoa Bioactive Compounds on Oxidative and Inflammatory Responses. Created in BioRender (https://biorender.com/pupd2rq accessed on 31 January 2026).

Cocoa has been present in human life since ancient times and was considered a food that could serve as an adjuvant in the treatment of diseases [63,85,86]. Cocoa contains several bioactive compounds, such as polyphenols and phenolic compounds, flavanols, anthocyanins, flavones, and flavanones. Among flavanols, (+)-catechin and (-)-epicatechin are the predominant bioactive monomers that have been associated with several cardiometabolic benefits [42,87,88,89]. Thus, some studies indicate that epicatechins in dark chocolate are associated with a reduced risk of mortality from CVD, MASLD, and cancer, in addition to their ability to stimulate brain function [62,90,91,92,93]. These compounds from cocoa are consequently present in chocolate (in different proportions, depending on the type of chocolate). They are known to enhance endothelial function and have anti-inflammatory properties, playing a crucial role in protecting the organism against CVD and MASLD, conditions that, as mentioned above, are associated with inflammatory and oxidative processes [94,95,96,97,98]. Figure 1 provides an overview of the antioxidant and anti-inflammatory effects of cocoa.

Due to the above, this narrative review uniquely integrates evidence on chocolate’s effects on both cardiovascular and hepatic health, exploring shared mechanisms and clinical implications. While previous reviews have focused on isolated systems, this analysis provides an integrative overview of chocolate’s role in the cardiometabolic continuum. A brief description of the literature selection approach is provided in Appendix A.

Although cocoa flavanols are present in several plant-based foods, chocolate represents a unique and widely consumed matrix in which these compounds are delivered alongside methylxanthines and lipids that may modulate their bioavailability and biological effects [62]. Moreover, chocolate consumption has been directly examined in a growing number of clinical trials assessing cardiovascular and hepatic outcomes, allowing a focused evaluation of this specific food rather than isolated bioactive compounds.

## 2. Chocolate Overview and Production

According to data published by the Observatory of Economic Complexity (OEC), global chocolate trade reached approximately USD 37 billion in 2023, up 10% compared to 2022. Over the last five years, trade in this category has grown at an annual rate of approximately 5%. Over time, chocolate became extremely popular worldwide, and the industry, upon recognizing the connection between the pleasure of consuming this food and nutritious eating, began to promote its benefits for well-being and basic nutritional health [99,100,101,102].

When evaluating the dry weight of the cocoa bean, proteins and their presence, such as albumin and globulin, which contain high levels of amino acids like glutamic acid, aspartic acid, lysine, and leucine, are observed [103,104]. The presence of these molecules is crucial for maintaining the quality of the final product (chocolate) [105].

Cocoa has been the subject of numerous studies in both agricultural and industrial contexts due to its economic relevance and the growing interest in high-quality products. The management practices applied throughout the entire production chain, from cultivation to chocolate manufacturing, directly influence the final product’s sensory and physicochemical characteristics [42,106].

Chocolate flavor profiles can vary significantly. Products with more complex and delicate sensory notes, such as fruity, floral, or caramel aromas, are highly valued in specialized and premium markets. In contrast, the functional food market tends to favor less processed versions with more intense flavors and natural formulations free of chemical additives [107,108].

These sensory variations are closely linked to factors such as cocoa variety, bean ripeness, agricultural practices, processing techniques, and environmental conditions—particularly geographic location, soil composition, rainfall patterns, solar exposure, and climate [37,53,109,110].

### Cocoa Processing Stages Leading to Chocolate Production

**Figure 2:**
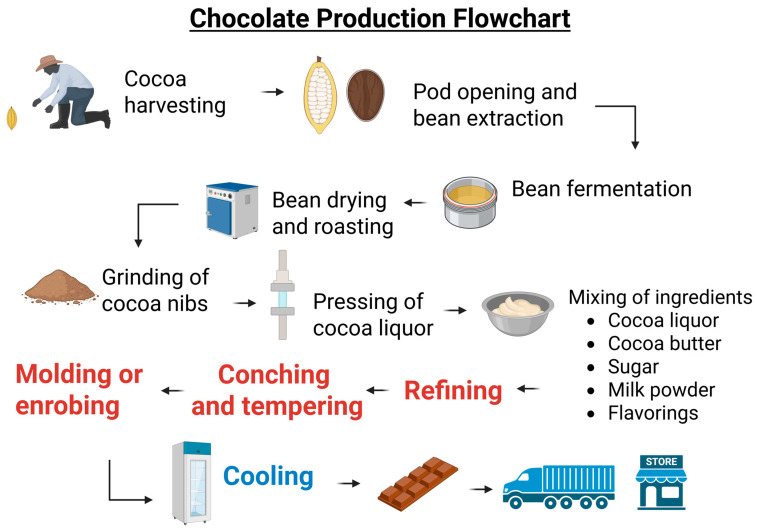
Flowchart for Chocolate Production. Created in BioRender (https://biorender.com/quthz1f accessed on 31 January 2026).

The transformation of cocoa into chocolate involves a sequence of steps designed to preserve and enhance its sensory attributes while ensuring product stability and quality [53,89,102,111,112,113,114]. Figure 2 shows the flowchart for chocolate production.

1. Cultivation and Harvesting: Cocoa plants are grown in tropical regions, and their fruits (called pods) are manually harvested at peak ripeness.
2. Fermentation: Once extracted, the seeds undergo fermentation, an essential step for developing the precursors of chocolate’s characteristic aroma and flavor.
3. Drying: The fermented seeds are sun-dried or dried by artificial methods to reduce moisture content and enable proper storage.
4. Roasting: Once dried, the seeds are roasted to enhance flavor and eliminate undesirable compounds.
5. Grinding: Roasted seeds are ground to produce cocoa mass, also known as cocoa liquor.
6. Refining: The cocoa mass is refined to achieve a fine and uniform texture, crucial for the product’s sensory quality.
7. Conching: This step involves continuous mixing and heating of the mass, which promotes the release of volatile acids and improves texture and flavor.
8. Tempering: The chocolate is subjected to precise temperature control to ensure the formation of stable cocoa butter crystals, resulting in a shiny appearance and firmness.
9. Molding and Cooling: The tempered chocolate is molded into the desired shapes and cooled until completely solidified.
10. Packaging, Storage, and Distribution: The product is then packaged and stored under controlled conditions to maintain its physicochemical and sensory properties.

It is important to note that these steps may vary depending on the type of chocolate produced (e.g., dark, milk, white, functional) and the specific standards adopted by each industry [115,116,117].

## 3. Chocolate Bioactive Compounds and Their Effects on Inflammation and Oxidative Stress

The health benefits of chocolate are attributed to the antioxidant properties of polyphenols found in the cocoa bean. It is one of the primary sources of dietary phenolics [118]. The main flavonoids in chocolate and cocoa beans are epicatechin, catechins, and procyanidins; procyanidins have potent antioxidant, anti-inflammatory, and cardioprotective properties [73]. Polyphenols are primarily detected in dark chocolate, with levels of approximately 12–15 mg/g [61,62]. When subjected to temperature changes, some compounds may undergo structural changes [110].

Chocolate also contains methylxanthines, such as theobromine and caffeine, which have central nervous system (CNS)-stimulating properties, as well as vasodilatory and diuretic effects [38,71,119,120]. Another group of bioactive compounds present in chocolate is biogenic amines, including phenylethylamine, histamine, and serotonin, which are generally associated with neuromodulatory effects, such as a sense of well-being, and vasopressor effects [121,122]. Bioactive peptides generated during the fermentation and digestion of cocoa proteins have also been identified as having antioxidant, antihypertensive, and anti-obesogenic effects [123]. It is important to note that chocolate also contains smaller amounts of phenolic acids and flavonols, all of which have potential antioxidant effects [73].

The various methods of cocoa processing significantly affect antioxidant levels, which can be influenced by factors such as geographic origin, cultivation, drying, fermentation, and industrial processing [124,125]. Cocoa and chocolate differ not only in their polyphenol profiles but also in how they are presented. Cocoa is sold in powder form, obtained by grinding the beans, while chocolate consists of a combination of cocoa, cocoa butter, sugar, and other ingredients, resulting in a solid product [62,73].

As discussed above, oxidative stress and inflammation are strongly associated with the development of chronic diseases, such as MetS, MASLD, and CVD risk factors [126,127]. This oxidative state is characterized by an imbalance between the body’s antioxidant defense capacity and the production of reactive oxygen species (ROS), including superoxide, hydroxyl, and hydrogen peroxide. These ROS, although essential for maintaining physiological processes (e.g., homeostatic redox balance), can activate pro-inflammatory signaling pathways and damage proteins, lipids, and cellular DNA [128,129,130]. The intake of antioxidant compounds helps neutralize free radicals and prevent oxidative reactions, thereby supporting endogenous antioxidant systems and protecting cellular DNA [131]. Cocoa is considered a superfood because of its high antioxidant potential, since it acts on oxidative stress [131,132,133,134].

Several studies have highlighted that the benefits of flavonoids present in cocoa and dark chocolate are linked to their specific physicochemical and structural characteristics. These attributes enable this class of compounds to modulate the expression and activation of pro-inflammatory cytokines, such as interleukin-1 beta (IL-1β), tumor necrosis factor-alpha (TNF-α) and interleukin-6 (IL-6), and control cellular apoptosis, the activity of ROS-scavenging enzymes and some crucial signaling pathways, such as the Bcl-2 family of proteins and nuclear factor erythroid 2-related factor 2 (Nrf2) [128,135]. The activation of specific receptors in cases of inflammation promotes the induction of intracellular pathways, including certain protein kinases and nuclear factor kappa-B (NF-κB), which are responsible for the signal transduction and the upregulation of pro-inflammatory cytokines, chemokines, and adhesion molecules [136,137,138]. Some researchers highlight the fact that flavonoids are excellent inhibitors of some inflammatory pathways, including the NF-κB pathway by blocking nuclear translocation, for example [136,139]. These characteristics give cocoa and, consequently, dark chocolate anti-inflammatory, neuroprotective, anticancer, cardioprotective, and metabolic-health promoting properties [128,140,141].

Consuming dark chocolate rather than white has been linked to positive effects on mood and depression, as dark chocolate contains higher cocoa content, less sugar, and superior antioxidant properties, thereby maximizing its benefits. Furthermore, there is evidence that cocoa in chocolate may improve sleep quality, potentially through mechanisms involving tryptophan and flavonoid content, modulation of circadian rhythms, and increased cerebral blood flow [73,142,143,144,145,146,147].

In addition to the metabolic and inflammatory mechanisms discussed above, gastrointestinal and hepatic inflammatory conditions themselves are increasingly recognized as contributors to cardiovascular risk. A narrative review by Ciccone et al. highlights the linkage between inflammatory bowel disease, hepatitis, non-alcoholic fatty liver disease, and early impairment of vascular endothelial function, which may predispose one to atherosclerosis and elevate cardiovascular risk beyond traditional factors [148]. These observations underscore that chronic systemic inflammation arising from liver and gastrointestinal disorders can interact with metabolic and vascular pathways, such as endothelial dysfunction and pro-atherogenic processes, complementing the shared pathophysiology central to cardiometabolic diseases such as MASLD and CVD.

### 3.1. Chocolate Consumption and Inflammation

Inflammation is a natural process that is carefully controlled by mechanisms that prevent excessive damage to adjacent tissues [149,150]. Some studies have demonstrated a relationship between dietary patterns rich in flavonoids, including cocoa and dark chocolate consumption, and anti-inflammatory effects [151,152,153,154,155]. The mechanisms by which some compounds in chocolate regulate inflammation are highly diverse. Some studies have shown that they can reduce levels of specific, highly important proinflammatory mediators, such as TNF-α, IL-6, interleukin-8 (IL-8), and Monocyte Chemoattractant Protein-1 (MCP-1), thereby corroborating a decrease in the inflammatory response [149,156,157,158]. Furthermore, such components have the ability to influence other signaling pathways that participate in inflammation, for example, through the inhibition of the enzymes cyclooxygenase-2 (COX-2) and lipoxygenase (LOX), as well as Activator Protein 1 (AP-1) and NF-κB, which consequently act on the activity of Mitogen-Activated Protein Kinase (MAPK) enzymes and interfere with the activation of some cells of the immune system [149,158].

### 3.2. Chocolate Consumption and Oxidative Stress

Oxidation is a natural process that enables vital reactions in organisms, such as cellular respiration and fatty acid metabolism. Cellular respiration occurs in the mitochondria and is responsible for the production of Adenosine Triphosphate (ATP) through a process called the respiratory chain. This process involves several mechanisms, including the oxidation of certain coenzymes and the transport of electrons. In this context, fatty acid metabolism occurs via β-oxidation, yielding acetyl-CoA, which feeds the tricarboxylic acid cycle and supports mitochondrial electron transport [159,160]. These oxidative processes generate ROS and harmful free radicals. Therefore, the body has an antioxidant system composed of enzymes, including catalase, glutathione peroxidase, and superoxide dismutase, which, together with its mitochondrial antioxidant system, can neutralize harmful oxidants and prevent excessive tissue damage [161,162,163,164,165]. The accumulation of free radicals is a process known as oxidative stress, characterized by damage to the structures of lipids, proteins, and nucleic acids [159,166].

Oxidative stress can be associated with the development of several diseases, as it contributes to the activation of proinflammatory pathways and mitochondrial dysfunction [166,167,168,169]. Both processes actively contribute to the progression of insulin resistance by increasing the production of Advanced Glycation End-products (AGEs), which aggravate the inflammatory milieu and oxidative stress, thereby impairing insulin signaling [168,170,171,172,173,174,175,176]. In this sense, studies indicate that eating dark chocolate attenuates the effects of oxidative stress and inflammation by inducing the activity of enzymes such as Glutathione S-transferase, Glutathione peroxidase, Glutathione reductase, superoxide dismutase, and catalase [149,177].

## 4. Chocolate Consumption, Obesity, and Glycemia

Chocolate may influence glycemic responses because it is primarily composed of carbohydrates (~50–60%) and is often consumed as a single snack [178].

Some studies suggest that chocolate consumption may be associated with relatively low postprandial glycemic responses, although these effects appear to vary according to chocolate composition. While insulin responses are generally comparable to those induced by reference foods, changes in glucagon-like peptide-1 (GLP-1) secretion have been inconsistent; nevertheless, polyphenol-rich chocolates have been linked to lower plasma glucose concentrations at later postprandial timepoints, potentially related to early insulin and incretin responses [179,180].

Studies suggest that polyphenols may influence pathways related to lipid metabolism and adipose tissue function, potentially through antioxidant and anti-inflammatory mechanisms [129,181,182].

Studies have demonstrated a protective effect on the intestinal barrier, where the authors used CaCo-2 cells (a model of the human intestinal mucosa) that were pretreated with different concentrations of theobromine and subsequently exposed to oxysterols [183]. This laboratory study enabled the observation of reduced cellular damage and decreased levels of inflammatory and pro-apoptotic markers. Other authors have demonstrated that mice treated with cocoa extract undergo a transformation from white to brown adipose tissue and exhibit improved glucose tolerance [184].

Some studies have reported modest improvements in cardiometabolic and oxidative stress markers following cocoa or cocoa-derived flavanol consumption. These effects are thought to be mediated primarily through antioxidant and anti-inflammatory mechanisms, as well as potential interactions with the gut microbiota; however, evidence regarding glycemic control, lipid metabolism, and insulin sensitivity in humans remains limited [144,185,186,187].

## 5. Chocolate Consumption and Lipids

It has been hypothesized that the ingestion of chocolate rich in cocoa may confer an additional beneficial effect on the endothelium through polyphenols, which may mediate increased nitric oxide (NO) bioavailability, thereby favoring blood flow [181,185,186].

The role of compounds present in cocoa products is not limited to the influence on lipid peroxidation. Consuming moderate amounts of dark chocolate can reduce Low-density lipoprotein cholesterol (LDL-c) and total cholesterol levels [62]. As mentioned above, dark chocolate affects inflammation and oxidative stress through several mechanisms of action. Moreover, it enhances the expression of mRNA of anti-inflammatory cytokines, such as interleukin-10 (IL-10), and is capable of modulating the action of immune cells (macrophages and other leukocytes); therefore, its antioxidant and anti-inflammatory compounds affect the lipid profile by decreasing the amount of oxidized LDL-c in the blood [62,93,188].

In addition to effects on circulating lipid concentrations, cocoa has been shown to influence lipid metabolism and lipid accumulation. Experimental evidence in animal models indicates that cocoa ingestion can modulate the expression of genes involved in fatty acid synthesis in liver and white adipose tissue and transport in white adipose tissue, leading to reduced white adipose tissue mass in high-fat-diet-induced obesity models. For example, cocoa supplementation in rats attenuated high-fat-diet-induced weight gain and visceral fat accumulation, decreased expression of genes related to fatty acid synthesis, and upregulated thermogenesis-related pathways in adipose tissue and liver, suggesting enhanced lipid utilization and reduced lipid storage [189].

Cocoa supplementation has also been shown to ameliorate diet-induced obesity, reduce adipocyte size, and decrease hepatic triglyceride accumulation, accompanied by downregulation of adipogenic genes and improvements in insulin sensitivity [190]. In vitro studies further demonstrate that cocoa polyphenol extracts can suppress adipogenesis and lipid accumulation in preadipocytes by inhibiting key differentiation pathways, including PPARγ and CEBPα signaling [191].

Furthermore, animal studies and mechanistic reviews suggest that flavanols may regulate lipid homeostasis by modulating lipid metabolism gene expression in the liver, reducing intrahepatic triglyceride content, and altering the balance between lipogenic and lipolytic pathways (e.g., upregulation of PPARα and downregulation of lipogenic enzymes), which can potentially attenuate excessive lipid deposition in metabolic tissues [192]. Collectively, these findings support the notion that cocoa flavanols may contribute to reduced lipid accumulation and improved lipid metabolism, mechanisms that are relevant to obesity and the development of metabolic disorders such as MASLD and CVD.

## 6. Chocolate Consumption and Hypertension

Hypertension is a major contributor to the global burden of disease, with prevalence increasing markedly [193,194]. Hypertension increases the risk of CVD and is one of the leading causes of premature death. Dark chocolate is rich in cocoa, which has been shown to promote antihypertensive effects [195,196]. A randomized controlled trial has shown that cocoa supplementation can improve endothelial function after injury [197]. Another study suggested that regular dietary consumption of 10 g of 99% cocoa dark chocolate in women over 6 months could be associated with a slight improvement in cardiovascular health [198]. Another study found that cocoa appears to lower blood pressure by increasing endothelial NO synthesis, promoting greater endothelium-dependent vasodilation, and inhibiting angiotensin-converting enzyme activity, thereby reducing blood pressure [92]. Dark chocolate has been associated with several health benefits, including the mitigation of CVD, reduction in blood pressure, and improvement of cognitive health [199]. Evidence suggests that diets rich in polyphenols, including those derived from dark chocolate, may be associated with benefits for psychological well-being and cardiovascular health, largely attributed to their flavanol content [200,201,202]. Chocolate may beneficially modulate cardiovascular responses under conditions of acute stress [203].

## 7. Chocolate Consumption and Endothelial Dysfunction

The endothelium produces vasoactive molecules, such as NO and endothelin. The imbalance in the production of these vasoactive substances leads to endothelial dysfunction, a condition characterized by a loss of function [204,205,206,207]. Endothelial dysfunction plays a crucial role in the development of atherosclerosis and can be triggered and exacerbated by various cardiovascular and cardiometabolic risk factors [208,209]. Analyzing the potential to improve endothelial dysfunction, a study using dark chocolate demonstrated its significant contribution to improving endothelial function, as evidenced by increased flow-mediated dilation (FMD) [98]. Additionally, chocolate-containing formulations promote artery dilation by reducing oxidative stress and increasing NO bioavailability. In particular, chocolate-containing formulations can enhance arterial dilatation by reducing nicotinamide adenine dinucleotide phosphate (NADPH) oxidase 2 (NOX2) activation, which has been shown in humans and animal models to exert vasoconstrictor activity [210]. Oxidative stress may cause functional disorders of the vascular endothelium, leading to endothelial apoptosis and alterations in vascular tissue structure and function. As noted above, studies have demonstrated that a cocoa polyphenolic extract can protect human endothelial cells against oxidative insult by modulating ROS generation and enhancing both enzymatic and non-enzymatic antioxidant defenses [211].

In situations of acute vascular challenge, such as eccentric exercise, the ingestion of microencapsulated cocoa attenuated the reduction in FMD, indicating a protective effect on vascular integrity [212]. Similarly, during episodes of mental stress combined with high-fat meals, both of which are known to impair endothelial function, the inclusion of high-flavanol cocoa mitigated the adverse effects and preserved endothelial vasodilatory responses [213]. Furthermore, when combined with dietary inorganic nitrate, which also promotes vasodilation, cocoa flavonoids showed additive effects on endothelial function, even at low doses. This suggests a synergistic potential for these dietary components to enhance vascular health [214].

**Figure 3:**
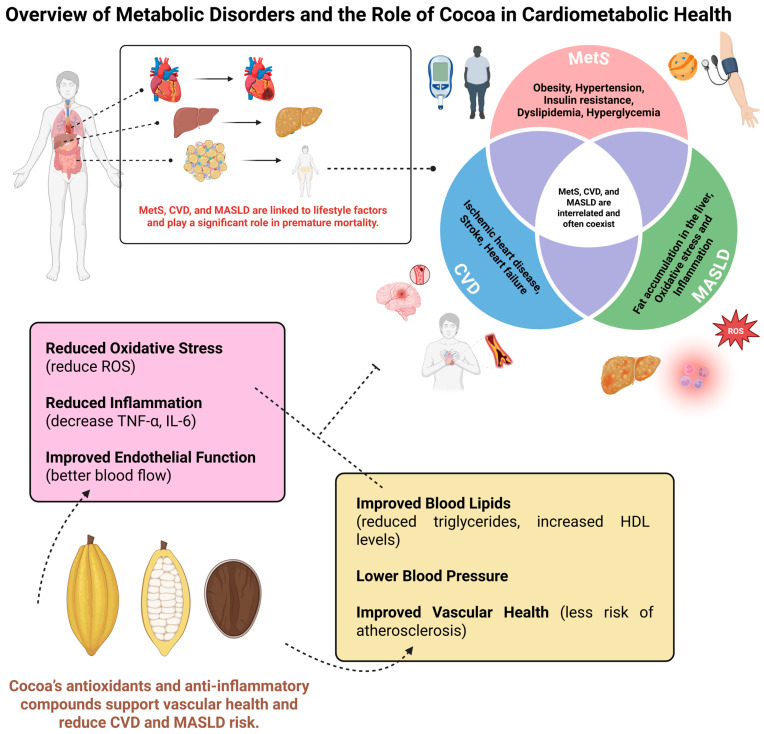
Effects of Cocoa on Cardiometabolic Health. Created in BioRender (https://biorender.com/8etd1nd accessed on 31 January 2026).

Figure 3 summarizes the effects of cocoa on cardiometabolic health.

## 8. Clinical Trials Showing the Effects of Chocolate Consumption on CVD and Related Risk Factors

Montagnana et al. [215] demonstrated that a single 50 g dose of 90% cocoa dark chocolate improved platelet function mediated by flavan-3-ol metabolites in healthy individuals. However, it lacks a control group.

Some authors conducted a one-week intervention with 30 g/day of 65% cocoa dark chocolate in patients with stable coronary artery disease undergoing dual antiplatelet therapy (aspirin + clopidogrel). The study showed a significant reduction in P2Y12 Reaction Units (PRU), indicating increased responsiveness to clopidogrel, without changes in aspirin effect. Together, these findings support the potential of dark chocolate to modulate platelet reactivity and contribute to cardiovascular protection. The short intervention period is a limitation of the study [216].

The randomized, crossover study performed by Dural et al. [98] evaluated patients with heart failure and reduced ejection fraction. This study assessed the relationship between chocolate consumption and endothelial dysfunction and found that dark chocolate improved FMD. However, the sample size was small.

Another trial investigated the effects of cocoa-rich chocolate on blood pressure and arterial stiffness in postmenopausal women. Only 3 of the 140 participants did not complete the study. The inclusion of a high-risk population group and the assessment of multiple vascular health parameters were strengths of the study. The difficulty of blinding participants due to chocolate’s sensory characteristics remains a potential source of bias [198].

Regecova et al. designed a randomized study to evaluate the acute effects of a single dose of dark chocolate in healthy young women. The outcomes were well-defined and measured using valid protocols. However, the study had a small sample size, which limited the generalizability of the findings. The observed effect may be transient and of uncertain long-term clinical significance [203].

The randomized controlled clinical trial by Leyva-Soto et al. has a long-term follow-up (6 months), demonstrating that flavonoid-rich chocolate reduces DNA damage and improves lipid parameters. However, one limitation is the small sample size, which limits the generalizability of the results [217].

One randomized, double-blind clinical trial evaluated the effects of chocolate high in flavanols and theobromine on endothelial function, blood pressure, and arterial stiffness in pregnant women at risk of preeclampsia. The study reported relevant clinical outcomes, focused on a unique and vulnerable population (pregnant women). Limitations include the inherent difficulty of controlling for all dietary and lifestyle variables during pregnancy, which may lead to residual confounding [218].

Overall, the study performed by Dicks et al. concluded that regular intake of flavanol-rich cocoa powder did not affect cardiometabolic parameters in patients with type 2 diabetes and hypertension. Its main strength is the double-blind, randomized, placebo-controlled design in a stable medication population, which provides a robust assessment of real-world efficacy. A limitation is that concomitant medications may have confounded the effects attributed to the intervention [219].

A randomized crossover trial was designed to compare the effects of dark chocolate, cocoa, almonds, and their combination in overweight/obese individuals. The main strength is the controlled-feeding design. Limitations include the short intervention period and the fact that the participants were not blinded to the interventions, as the food provided was easily identifiable, which exposes the study to expectation bias [220].

The study by Hammer et al. was a randomized, controlled, crossover clinical trial investigating the effects of dark chocolate on vascular function in patients with peripheral artery disease. However, a combination of factors characterizes potential biases, and it is relevant to note: the small sample size (n = 21), the imbalance in the group’s composition (17 men and four women), and the fact that the dark chocolate intervention comprised a single administration [221].

A double-blind, randomized clinical trial compared high- and low-flavanol chocolate in men with prehypertension or mild hypertension. The outcomes were objective and included vascular function and platelet aggregation assessments. Losses were minimal, and participant characteristics were well described. A limitation is the small sample size. Another bias is the use of a specific commercial product, which may raise questions about the reproducibility of the results with other chocolate sources [222].

Koli et al. designed a crossover intervention study involving adults with mild hypertension, in which participants replaced snacks with dark chocolate. However, limitations include a small sample size and the lack of a control product [223].

Taken together, clinical trials evaluating chocolate consumption and cardiovascular outcomes suggest that potential benefits are highly context-dependent and largely confined to intermediate vascular and platelet biomarkers rather than hard clinical endpoints. Improvements in endothelial function, platelet reactivity, and lipid-related parameters are more consistently reported in short-term interventions, in relatively small samples, and in populations without advanced CVD or extensive pharmacological treatment. In contrast, studies conducted in patients with established cardiometabolic conditions or under stable multidrug regimens frequently report null or modest effects, indicating a limited incremental benefit beyond standard therapy.

Methodological heterogeneity represents a major challenge to interpretation. Studies vary substantially in cocoa content, flavanol dose, duration of exposure, and form of administration (solid chocolate vs. cocoa products), limiting direct comparability across trials. Furthermore, many interventions are characterized by small sample sizes, short follow-up periods, and crossover designs that, while efficient, may be insufficient to capture sustained vascular adaptations or long-term cardiovascular risk modification. The frequent difficulty in achieving adequate blinding due to chocolate’s sensory properties introduces an additional risk of expectation and performance bias.

Importantly, positive findings are predominantly observed in surrogate markers such as FMD, platelet aggregation indices, and arterial stiffness, which, although biologically relevant, do not necessarily translate into clinically meaningful reductions in cardiovascular events. The inconsistent effects on blood pressure, glycemic control, and lipid profiles across studies further underscore the need for cautious interpretation. Overall, the current evidence supports a plausible mechanistic role for cocoa flavanols in vascular health, but remains insufficient to substantiate definitive cardiovascular protection or to inform clinical recommendations.

These studies are presented in Table 2.

## 9. Clinical Trials Showing the Effects of Chocolate Consumption on MASLD-Related Risk Factors

MASLD is a liver disease characterized by an abnormal accumulation of lipids in the liver and, as a consequence of several previous modifications in the organism, changes in lipid patterns and inflammatory states [152]. Thus, it is possible to conclude that progressive lipid accumulation associated with increasing inflammation and oxidative stress contributes to the emergence of the disease [224]. Chocolate consumption can be considered a means to improve MASLD risk factors, given its previously mentioned properties [154,157,225,226,227].

Pannunzio et al. used a randomized, crossover design with 19 patients diagnosed with MASH, comparing dark chocolate (>85% cocoa) with milk chocolate (<35% cocoa) over 14 days. They evaluated endotoxemia, a phenomenon linked to systemic inflammation, in these patients. The primary strength of this study lies in its investigation of a specific, innovative pathophysiological mechanism underlying the MASH. However, it was not double-blinded, which increases the risk of bias [228].

Mancin et al. investigated whether ingesting 30 g of dark chocolate for 4 weeks is associated with a significant improvement in the blood lipid profile, resulting in reductions in LDL-c, triglycerides, and total cholesterol in a randomized controlled trial. The main limitations were a short intervention period (four weeks) and the exclusion of women. It is important to note that 38 randomized subjects completed the intervention [97].

Wiese et al. aimed to investigate the combined prebiotic effect of lycopene and dark chocolate on the gut microbiome and consequences on liver metabolism, muscle, and skin in participants with moderate obesity. A benefit of the study is its integrated translational approach, which connects gut health to the health of other organs. However, the study included a relatively small sample size (n = 30), which may limit the generalizability of the results. Moreover, the intervention period was relatively short, only one month, insufficient to assess sustained changes in clinical and microbiological parameters. Taken together, these limitations may have influenced the reliability of the results reported in the article [229].

Loffredo et al. performed a randomized crossover intervention with 19 patients with non-alcoholic steatohepatitis (NASH). Participants consumed dark chocolate (>85% cocoa) or milk chocolate (<35% cocoa) for 14 days. Outcomes included FMD, isoprostanes, and NO. The outcomes were measured using reliable laboratory methods. However, it was not double-blinded, which limits internal validity [91].

Another trial used a randomized crossover design with 19 patients with NASH and 19 healthy controls. Participants consumed either dark or milk chocolate for two weeks, and outcomes included NOX2 activity, isoprostanes, and hepatocyte apoptosis (as indicated by CK-18). The outcomes were measured using validated laboratory methods. However, it was not double-blinded, and the sample size was small, limiting statistical reliability [230]. 

Altogether, these findings reinforce the notion that incorporating flavonoid-rich foods, such as dark chocolate, into high-quality dietary patterns may be an effective strategy to mitigate cardiovascular and MASLD risk.

Clinical trials investigating the effects of dark chocolate on MASLD and related metabolic disturbances are limited in number and predominantly exploratory. Most available studies employ small sample sizes, short intervention periods, and crossover designs, which restrict the ability to draw firm conclusions regarding sustained hepatic or metabolic benefits. While reductions in markers of oxidative stress, endotoxemia, and endothelial dysfunction have been reported, these outcomes largely reflect intermediate or mechanistic endpoints rather than direct measures of liver fat content, fibrosis progression, or long-term clinical outcomes.

A recurring strength of this body of literature is the mechanistic focus on pathways linking gut permeability, systemic inflammation, oxidative stress, and hepatic injury. However, the lack of double-blinding in several trials, combined with the short duration of interventions, raises concerns regarding internal validity and the persistence of observed effects. Moreover, many studies involve highly selected populations—such as male athletes, patients with early-stage disease, or individuals without significant comorbidities—thereby limiting external validity and generalizability to the broader MASLD population.

Although the findings collectively suggest that cocoa flavanol-rich dark chocolate may favorably modulate cardiometabolic and inflammatory pathways implicated in MASLD, the evidence remains insufficient to support its use as a therapeutic or preventive strategy. Rather, current data should be interpreted as hypothesis-generating, supporting further investigation of flavonoid-rich foods within comprehensive dietary patterns, ideally through larger, longer-term, well-controlled trials assessing clinically relevant hepatic endpoints.

These studies are presented in Table 3.

## 10. Heterogeneity of Results and Modulating Factors

Despite the growing body of evidence supporting the benefits of dark chocolate, conflicting results deserve critical consideration. Studies such as those by Dicks et al. [219] in medicated diabetic and hypertensive patients, and Hammer et al. [221] in patients with established peripheral artery disease, failed to demonstrate significant benefits. This heterogeneity may reflect several factors: first, the window of opportunity for nutritional interventions may be more favorable in the early stages of metabolic dysfunction, before irreversible structural changes occur. Second, polypharmacy in populations with chronic diseases may mask or inhibit the additive effects of nutritional interventions. Third, there are issues of dosage and bioavailability—many studies employ commercial products with significant variability in flavanol content.

It is important to note that the lack of effect in specific populations does not invalidate the benefits in prevention settings or at earlier stages of disease. Instead, it highlights the need for personalized recommendations and better characterization of biomarkers of response.

In summary, we suggest that the conflicting results in the literature can be understood through different perspectives.

The benefits of these interventions may be more evident in primary prevention and in the early stages of disease than in established chronic conditions. Nutritional interventions tend to be more effective when implemented during the initial phases of cardiometabolic pathophysiology or liver disease progression. Moreover, dose-response relationships appear to play a critical role, as studies reporting negative outcomes often employ lower doses or formulations with reduced bioavailability of active compounds. Finally, discrepancies among studies may also be attributed to differences in the amount of chocolate consumed and the timing of supplementation.

An important limitation of the current evidence base is that many clinical studies investigating the effects of chocolate or cocoa-derived products have small sample sizes and limited statistical power. Small sample sizes, combined with short intervention durations and heterogeneous study designs, reduce the precision of estimated effects and increase the risk of type II error, making it difficult to draw definitive conclusions about the clinical significance of observed outcomes.

As a result, while some studies report beneficial effects on biomarkers such as endothelial function, lipid profiles, and markers of oxidative stress, the small number of participants and variability across trials temper confidence in these findings. Larger randomized controlled trials with adequately powered sample sizes are therefore necessary to validate the preliminary effects observed to date and to establish more definitive evidence for the role of cocoa flavanols and dark chocolate consumption in cardiometabolic health.

An additional methodological concern is the difficulty of achieving effective blinding in chocolate-based interventions, given their sensory characteristics, which may introduce expectation bias. Furthermore, the frequent use of specific commercial products limits reproducibility and generalizability.

## 11. Conclusions and Limitations

The findings of this review suggest that regular and moderate consumption of dark chocolate, particularly varieties rich in cocoa flavanols, may have significant beneficial effects on cardiovascular and hepatic health. The bioactive compounds in cocoa exhibit potent antioxidant, anti-inflammatory, and vasoprotective properties, which can modulate molecular pathways involved in oxidative stress and endothelial dysfunction. Clinical evidence demonstrates improvements in lipid profile, blood pressure, endothelial function, and markers of liver injury and oxidative stress, suggesting a promising role for dark chocolate in reducing CVD and MASLD risk factors. However, most available clinical trials had small sample sizes and short intervention durations, limiting the ability to assess long-term effects. Additionally, the lack of standardized flavonoid dosages across studies and the use of heterogeneous commercial chocolate products with variable cocoa content make it difficult to establish reproducible outcomes. The sensory characteristics of chocolate also hinder proper blinding in randomized controlled trials, further increasing the risk of bias.

While the available evidence suggests that dark chocolate may exert favorable effects on intermediate cardiometabolic and hepatic biomarkers, the current data are insufficient to support firm clinical recommendations. Most findings should be interpreted as hypothesis-generating, given the predominance of small, short-term, and heterogeneous trials.

From a population perspective, most studies focused on specific groups, such as men, postmenopausal women, obese individuals, or athletes, thereby reducing the generalizability of results to the broader population. Evidence is particularly scarce in high-risk cohorts, including patients with advanced MASLD, severe hypertension, or type 2 diabetes. Furthermore, most studies were conducted in Western or European populations, with limited ethnic and geographic diversity.

Another relevant limitation lies in the lack of strict dietary control during interventions, which may confound the effects attributed to dark chocolate. In populations with chronic diseases, polypharmacy may also attenuate or obscure potential benefits. Moreover, studies assessed a wide range of heterogeneous outcomes (lipid profiles, blood pressure, hepatic markers, and inflammatory mediators), making direct comparisons and meta-analyses challenging.

Finally, there are practical limitations for clinical application. The optimal dose–response relationship of cocoa flavanols remains uncertain, and excessive chocolate intake may contribute to caloric overload, increased sugar consumption, and higher saturated fat intake, thereby counteracting the potential health benefits. Notably, the lack of large-scale, long-term randomized controlled trials prevents definitive conclusions about dark chocolate’s ability to reduce the incidence of cardiovascular or hepatic clinical events.

Future randomized controlled trials with larger cohorts, longer follow-up, and standardized cocoa flavanol dosages are essential to confirm the therapeutic potential of chocolate and to define optimal intake levels for clinical practice. Nonetheless, the current evidence suggests that dark chocolate can be considered a functional food, capable of contributing to cardiometabolic health when consumed responsibly as part of a balanced diet. Moreover, our integrative approach reveals how chocolate’s benefits extend beyond individual organ systems, offering novel insights for the holistic management of cardiometabolic and liver diseases.

## 12. Future Perspectives

Although promising, current evidence on the cardiometabolic benefits of chocolate is limited by methodological variability and the use of short-term interventions. Future research should prioritize large-scale, double-blind randomized controlled trials using standardized cocoa flavanol doses and formulations to ensure reproducibility and comparability among studies. It is also crucial to explore the dose–response relationship and to identify threshold levels at which chocolate provides optimal health benefits without promoting caloric excess. In summary, we recommend conducting large-scale trials in early disease stages, investigating standardized flavanol formulations and dosing regimens, evaluating long-term interventions, and implementing personalized nutrition approaches.

Advanced omics-based approaches (metabolomics, proteomics, lipidomics, and transcriptomics) may help elucidate the precise molecular targets of cocoa flavanols and their impact on oxidative stress, inflammatory mediators, and endothelial function. Furthermore, studies integrating gut microbiota analysis could clarify how cocoa polyphenols influence host metabolism and systemic inflammation through the gut–liver–heart axis.

Given the growing prevalence of CVD and MASLD, incorporating dark chocolate as a nutritional adjunct may represent an appealing and accessible preventive strategy. Interdisciplinary investigations that combine clinical nutrition, molecular biology, and pharmacology are essential for translating these findings into personalized nutrition approaches and potential nutraceutical applications to improve cardiovascular and hepatic outcomes.

The media also plays a role in informing the public that the health effects of chocolate vary depending on its type and that excessive consumption can be harmful due to high sugar and/or saturated fat levels.

### Practical Implications for Daily Practice

Evidence from prospective cohort studies suggests that moderate consumption of chocolate—particularly dark chocolate—is associated with a lower risk of coronary heart disease, stroke, and type 2 diabetes than low or no consumption, with little additional benefit beyond ~3–6 servings per week [231]. In large US cohorts, participants consuming ≥5 servings/week of dark chocolate had a significantly lower risk of incident type 2 diabetes [232]. A meta-analysis of randomized trials in patients with diabetes also reported improvements in LDL-c and fasting glucose after cocoa/dark chocolate consumption [233]. Although intervention evidence remains limited, mechanistic reviews support beneficial effects of cocoa flavanols on blood pressure, endothelial function, and insulin resistance, which may underlie cardiometabolic benefits [234].

## Figures and Tables

**Table 1:** Main bioactive compounds of cocoa and chocolate.

Bioactive Compound	Structure	General Effects on Humans	Reference
[Flavonoids] (Flavan-3-ols) Catechin	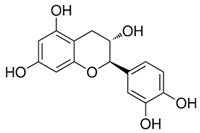	Antioxidant Anti-inflammatory Anti-cancer Blood pressure reduction Neuroprotection Improves insulin actions	[46,47,48,49,50,51,52,53,54,55]
[Flavonoids] (Flavan-3-ols) Epicatechin	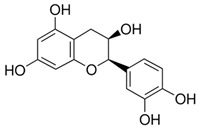	Antioxidant Anti-inflammatory Anti-cancer Blood pressure reduction Neuroprotection Improves insulin actions	[48,51,52,54,55,56,57,58]
[Flavonoids] (Flavan-3-ols) Procyanidins	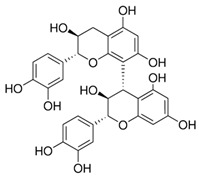 Procyanidin B3	Antioxidant Anti-inflammatory Anti-cancer Neuroprotection Improves insulin sensitivity	[46,48,49,51,52,54,55,59,60,61]
[Flavonoids] (Anthocyanidins) Cyanidin	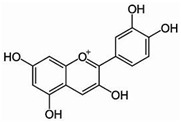	Antioxidant	[62,63]
[Flavonoids] (Flavonols) Quercetin and Isoquercetin	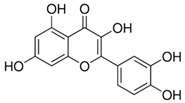 Quercetin 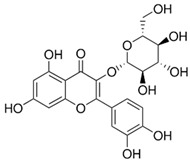 Isoquercetin	Antioxidant Anti-inflammatory Anti-cancer Cardiovascular protection Neuroprotection	[64,65,66,67,68,69]
[Phenolic Acids] Caffeic acid and Chlorogenic acid	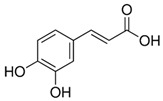 Caffeic acid 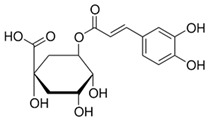 Chlorogenic acid	Antioxidant Anti-inflammatory Anticancer Antimicrobial	[51,53,69,70]
[Methylxanthine] Theobromine	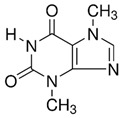	Vasodilation Cardiovascular protection Neuroprotection Bronchodilation Anticancer	[50,51,64,71,72,73,74]
[Methylxanthine] Caffeine	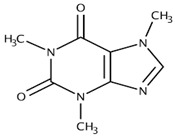	Vasodilation Cardiovascular protection Neuroprotection Anticancer	[50,51,53,71,72,74,75,76]
[Bioactive amine] Tryptamine and Tyramine	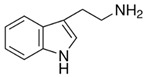 Tryptamine 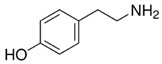 Tyramine	Neurological modulation Antioxidant Cardiovascular protection Mood modulation	[42,77,78,79,80,81]
[Phytosterols] β-sitosterol and Stigmasterol	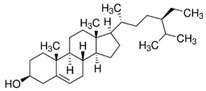 β-sitosterol 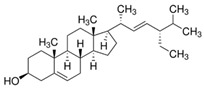 Stigmasterol	Anticancer Cardioprotection Improve lipid profile	[42,82,83,84]

**Table 2:** Studies showing the effects of chocolate on cardiovascular diseases and related risk factors.

Reference	Type of Study/Population/ Country	Intervention/Comparison	Outcomes	Adverse Effects
[98]	- Randomized crossover clinical trial; - 20 patients with heart failure and reduced EF < 40% (58.4 ± 9.2 y) (12 males and 8 females); - Country: Turkey.	- DC (80% cocoa) or MC (29% cocoa); - Duration: 2 weeks per phase with a wash-out period.	- NT-proBNP decreased significantly after both chocolates; - FMD improved after DC (~8.9% → ~14%, *p* = 0.019); - Catechin and epicatechin levels increased more after DC; - Negative correlation between catechin/epicatechin and NT-proBNP in both chocolates.	NR
[216]	- Prospective, open-label, pilot study; - 20 patients with stable coronary artery disease on dual antiplatelet therapy (aspirin + clopidogrel); - Age: ~61.4 y; - 13 males/7 females; - Trinidad y Tobago.	30 g/day of 65% cocoa DC for 7 days.	- ↓ PRU by 26.85 units (*p* = 0.001); - ↑ clopidogrel responsiveness; no change in aspirin effect.	No patients experienced any serious adverse events.
[198]	- Randomized, controlled parallel clinical trial; - 140 postmenopausal women (50–64 y), divided in two groups: IG (n = 73) and CG (n = 67); - Country: Spain.	- IG = 10 g of chocolate/daily (99% cocoa) and CG = no intervention; - 6 months.	A slight improvement in cardiovascular health (a decrease in pulse pressure) is possibly related to the intervention.	NR
[203]	- Randomized trial; - 42 healthy young female participants; - Country: Slovak Republic.	- DC (85% cocoa) or MC; - Single dose: 1 g/kg body weight.	- Resting: increase in SBP and double product after DC; - During mental stress: DC buffered the reactivity of DBP, HR, MAP, and double product; - MC showed no significant effects.	NR
[215]	- Interventional study with 18 healthy male volunteers; - Country: Italy; - Age: 36 ± 10 y.	- 50 g of 90% cocoa DC consumed once; - No control group.	- ↑ COL/ADP-induced PFA-100 closure time; - ↑ plasma flavan-3-ol metabolites; improved platelet function.	NR
[217]	- Randomized, placebo-controlled, double-blind study conducted in the USA with 47 men and 37 women; - Young volunteers with cardiovascular and MetS risk factors (age: 20–35 y).	- 42 subjects were allocated into IG = daily consumption of 2 g of DC (70% cocoa), and 42 were allocated into PG = daily consumption of 2 g of MC; - 6 months.	DC improved triglycerides, LDL-c and total cholesterol; the consumption could also be related to a decrease in cellular stress.	NR
[218]	- Randomized, double-blind, parallel-group trial; - 131 pregnant women at risk of preeclampsia; - Age: 18–38 y; - Single center: Canada.	- HFHT: 40 g (acute) and 30 g/day for 12 weeks (chronic); - LFLT: 40 g (acute) and 30 g/day for 12 weeks (chronic).	- Acute: compared to LFLT, HFHT increased epicatechin and theobromine levels and reduced arterial stiffness, with no effect on endothelial function; - Chronic: compared to LFLT, HFHT increased theobromine, but it did not have positive impacts on endothelial function, arterial stiffness or BP.	Nausea and digestive discomfort reported.
[219]	- Double-blinded, randomized, placebo-controlled trial with 35 patients (18 men and 17 women) with DM2 and hypertension; - Age: 64.2 ± 1.5 y; - Country: Germany.	The subjects were allocated into group A: received capsules with 2.5 g/day of flavanol-rich cocoa, and group B: received a placebo capsule (microcrystalline cellulose) daily for 12 weeks.	- No significant differences were detected in BP, glucose metabolism, and lipid profile in any group; - The results on outcome markers of intention-to-treat analysis (n = 42) were not different from those of the per-protocol analysis (n = 35).	NR
[220]	Randomized controlled, 4-period, crossover, feeding trial was conducted in the USA with 48 overweight and obese participants (30–70 y).	- Subjects consumed each of 4 diets: NTF (average American diet), ALD (42.5 g/daily of almonds), CD (43 g/daily of DC or 18 g/daily of cocoa powder) and all 3 foods (ALD and CD); - Each diet period lasted 4 weeks, followed by a 2-week compliance break.	The consumption of almonds and cocoa/DC had beneficial effects on LDL-c, Apolipoprotein B and lipid profile, which can be related to a reduced risk of coronary heart disease.	NR
[221]	Investigator blinded, randomized, controlled, cross-over trial conducted in Switzerland with 21 patients (17 men and 4 women) with symptomatic (Fontaine stage II) PAD.	Subjects were allocated into CG = 50 g of WC, and TG = 50 g of DC.	A single consumption of DC had no detected effect on microvascular function and endothelial function.	NR
[222]	- Randomized, double-blind, placebo-controlled crossover trial; - Single-center: United Kingdom; - 32 male participants (26 completed both phases); - Age: 45–70 y; - Condition: Pre-hypertension or mild hypertension.	- HFDC: 1064 mg flavanols/day (50 g/day); - LFDC: 88 mg flavanols/day (50 g/day); - Duration: 6 weeks per phase; - Frequency: twice daily (25 g morning, 25 g afternoon).	- HFDC showed modest improvements in cardiovascular function; - HR remained stable with HFDC, increased with LFDC; - Enhanced vascular response to salbutamol after HFDC; - Both chocolates reduced responses to ADP and TRAP6 relative to baseline.	NR
[223]	Randomized, controlled, and 8-week crossover study with 22 patients (8 women and 14 men) (aged 33–64 y) with mild hypertension.	- Intervention period: reduced habitual snack consumption and replaced them with DC (49 g/day); - Control period: reduced snacking only; - There was a wash-out period between both.	DC had no effects on 24 h BP, resting BP, and arterial stiffness; however, the BP decreased over the entire study.	NR

Abbreviations. DC: Dark Chocolate; MC: Milk Chocolate; NR: Not Reported; EF: Ejection Fraction; NT-proBNP: N-terminal pro b-type Natriuretic Peptide; FMD: Flow-Mediated Dilation; PRU: P2Y12 Reaction Units; IG: intervention group; CG: control group; SBP: Systolic Blood Pressure; DBP: Diastolic Blood Pressure; HR: Heart Rate; MAP: Mean Arterial Pressure; COL/ADP: Collagen/Adenosine Diphosphate; PFA-100: Platelet Function Analyzer-100; USA: United States of America; LDL-c: Low-density lipoprotein cholesterol; PG: Placebo Group; HFHT: High Flavanol High Theobromine Chocolate; LFLT: Low Flavanol Low Theobromine Chocolate; BP: Blood Pressure; DM2: Diabetes Mellitus type II; NTF: No Treatment Food; ALD: Almond Diet; CD: Chocolate Diet; PAD: Peripheral Artery Disease; WC: White Chocolate; TG: Treatment Group; HFDC: High Flavanol Dark Chocolate; LFDC: Low Flavanol Dark Chocolate; ADP: Adenosine Diphosphate; TRAP6: Thrombin Receptor Activator Peptide 6; MetS: Metabolic Syndrome.

**Table 3:** Studies showing the effects of chocolate on MASLD-related risk factors.

Reference	Type of Study/Population/Country	Intervention/Comparison	Outcomes	Adverse Effects
[228]	- Randomized, crossover, single-blind; - The study recruited 19 patients with MASH; - 11 males and 8 females (mean age 46.2 ± 11.2 y); - Country: Italy.	Participants consumed 40 g/day of chocolate, divided into 20 g every 12 h, for 14 consecutive days. The study occurred in two phases: DC (>85% cocoa solids) and MC (<35% cocoa solids), each followed by a minimum washout period of 1 week between the phases.	- A significant difference was observed between treatments in LPS (*p* = 0.04) and zonulin (*p* = 0.02); - After 14 days of DC, LPS levels decreased from 22 ± 4 to 19 ± 4 pg/dL (−15%), and zonulin levels decreased from 3.2 ± 0.9 to 2.5 ± 0.8 pg/mL (−20%).	NR
[97]	- A randomized controlled trial in a cohort of 38 elite male soccer players (27 ± 4 y); -Country: Italy.	- The subjects were randomly divided into two groups: DC group (n = 19) ingested 30 g of 88% cocoa DC; WC group (n = 19) was provided with 30 g of WC; - Each group ingested the chocolate intervention as a “solid bar” in the morning every day for 4 weeks.	- DC group showed increased plasma polyphenols (from 154.7 ± 18.6 μg gallic acid equivalents/mL to 185.11 ± 57.6 μg gallic acid equivalents/mL, Δ pre vs. post = +30.41 ± 21.50); - DC group also showed significant improvements in lipid profiles (Total cholesterol, triglycerides, and LDL-c) compared with WC group; - Significant increase in HDL-c in the group that consumed DC.	NR
[229]	- 30 volunteers (15 women and 15 men) (age: 55 ± 5.7 y) with moderate obesity; - The trial was double-blinded for the three lycopene groups and separately for the 2 DC groups (received blinded DC products); - Country: Denmark, United Kingdom, and Russia.	The subjects were divided into 5 groups and the trial lasted 1 month: 1st group → 10 g of DC with 7 mg lycopene/day; 2nd group → 7 mg GAL-MSFA (1 capsule/day); 3rd group → 30 mg GAL-MSFA (1 capsule/day); 4th group → 30 mg GAL-PUFA (1 capsule/day); 5th group → 10 g of the control DC/day.	DC with or without lycopene had a similar effect on the inhibition of inflammatory oxidative damage as 7 mg of lycopene. Although both chocolate products were able to reduce LDL-Px, their effectiveness was lower than that of lycopene.	NR
[91]	- Randomized, crossover, single-blind clinical trial; - 19 patients with NASH; - Mean age: 46 ± 11 y; - Country: Italy.	- All participants consumed 40 g/day of chocolate (20 g every 12 h) for 14 days; - Two phases: DC (>85% cocoa) and MC (<35% cocoa), separated by a 7-day washout.	Compared to baseline, FMD and NOx increased in subjects given DC but not in those given MC. A simple linear regression analysis showed that Δ (expressed by difference in values between before and after 14 days of chocolate assumption) of FMD was associated with Δ of NOX2 activity (Rs = −0.323; *p* = 0.04), serum isoprostanes (Rs: −0.553; *p* < 0.001) and NOx (Rs: 0.557; *p* < 0.001).	NR
[230]	- Randomized, single-blind, crossover study; - 19 patients with NASH; - Mean age: 46 ± 11 y; - Country: Italy.	Patients were allocated to groups of 40 g/day of DC (>85% cocoa) or 40 g/day of MC (<35% cocoa), for 2 weeks.	Compared to baseline, the intake of DC significantly reduced sNOX2-dp, serum isoprostanes and CK-18 levels. No change was observed after MC ingestion.	NR

Abbreviations. MASH: Metabolic dysfunction-associated steatohepatitis; DC: Dark chocolate; MC: Milk chocolate; LPS: Lipopolysaccharide; NR: Not Reported; WC: white chocolate; HDL-c: High Density Lipoprotein Cholesterol; LDL-c: Low Density Lipoprotein Cholesterol; GAL-MSFA: GA Lycopene formulated with Medium Saturated Fatty Acids; GAL-PUFA: GA Lycopene formulated with Polyunsaturated Fatty Acids; LDL-Px: LDL peroxidase; NASH: Non-alcoholic steatohepatitis; FMD: Flow-mediated dilation; NOx: Nitric oxide bioavailability; NOX2: NADPH oxidase 2; sNOX2-dp: Soluble NOX2-derivative peptide; CK-18: Cytokeratin-18.

## Data Availability

No new data were created or analyzed in this study. Data sharing does not apply to this article.

## Appendix A

### Approach to Literature Selection

A narrative literature review was conducted using the PubMed, Web of Science, and Scopus databases. Studies published in English, relevant to chocolate and the cardiometabolic continuum, including clinical trials, observational studies, and reviews, were considered. Titles and abstracts were screened to select articles for full-text review. This approach aimed to provide a representative and up-to-date overview of the topic rather than an exhaustive systematic synthesis.
